# Incorporating exposure to pitch canker disease to support management decisions of *Pinus pinaster* Ait. in the face of climate change

**DOI:** 10.1371/journal.pone.0171549

**Published:** 2017-02-13

**Authors:** María Jesús Serra-Varela, Ricardo Alía, Javier Pórtoles, Julián Gonzalo, Mario Soliño, Delphine Grivet, Rosa Raposo

**Affiliations:** 1 University of Valladolid, Department of Plant Production and Forest Resources, Palencia, Spain; 2 Sustainable Forest Management Research Institute, INIA- University of Valladolid, Palencia, Spain; 3 INIA, Forest Research Centre, Madrid, Spain; 4 Fundación para la Investigación del Clima, Madrid, Spain; Georg-August-Universitat Gottingen, GERMANY

## Abstract

Climate change is gravely affecting forest ecosystems, resulting in large distribution shifts as well as in increasing infection diseases and biological invasions. Accordingly, forest management requires an evaluation of exposure to climate change that should integrate both its abiotic and biotic components. Here we address the implications of climate change in an emerging disease by analysing both the host species (*Pinus pinaster*, Maritime pine) and the pathogen’s (*Fusarium circinatum*, pitch canker) environmental suitability i.e. estimating the host’s risk of habitat loss and the disease`s future environmental range. We constrained our study area to the Spanish Iberian Peninsula, where accurate climate and pitch canker occurrence databases were available. While *P*. *pinaster* is widely distributed across the study area, the disease has only been detected in its north-central and north-western edges. We fitted species distribution models for the current distribution of the conifer and the disease. Then, these models were projected into nine Global Climate Models and two different climatic scenarios which totalled to 18 different future climate predictions representative of 2050. Based on the level of agreement among them, we created future suitability maps for the pine and for the disease independently, which were then used to assess exposure of current populations of *P*. *pinaster* to abiotic and biotic effects of climate change. Almost the entire distribution of *P*. *pinaster* in the Spanish Iberian Peninsula will be subjected to abiotic exposure likely to be driven by the predicted increase in drought events in the future. Furthermore, we detected a reduction in exposure to pitch canker that will be concentrated along the north-western edge of the study area. Setting up breeding programs is recommended in highly exposed and productive populations, while silvicultural methods and monitoring should be applied in those less productive, but still exposed, populations.

## Introduction

Anthropogenic climate change affects forest ecosystems greatly, demanding management plans aimed at increasing the capacity of forests to cope with climate change, and guaranteeing that they maintain their essential role of providing services for society [1]. In this context, assessing exposure—an evaluation of the magnitude of climate change [2]—becomes crucial, and should ideally consider both abiotic and biotic factors [3]. The alterations of abiotic factors due to climate change, such as increased intensity and duration of droughts (mid-latitudes) and ascending global mean temperatures [4], have led to increased tree mortality [5] and northwards shift of distributions of many species [6]. Because of these major climate-related alterations, species-specific abiotic exposure has been commonly addressed in the literature [7–9]. In contrast, there have been less studies addressing how pests and pathogens are responding to climate change and their effect on hosts [10–14]. Biotic exposure is rarely contemplated when assessing vulnerability to climate change (some exceptions [15,16]), despite their effects becoming progressively evident [12,17]. Specifically, forest pathogen invasions have grown exponentially in Europe in the last decades, with introductions mainly from North America and recently from Asia [18]. In most situations, these invasions had an important consequence on native tree species: as Dutch elm disease on mature elm trees (*Ulmus minor*) in the 1970s [19], or ash dieback on ash (*Fraxinus excelsior*) since the 1990s [20].

Species distribution models (SDMs) [21,22]; provide a useful tool for assessing abiotic and biotic exposures, as they utilize associations between environmental variables and known species’ occurrence records [23], enabling the assessment of habitat suitability for a given species under different climatic scenarios [24]. This approach can also be extended to a host-disease relationship, by analyzing environmental suitability in each of the two components, and assuming that their interaction will not change. The relationship between environmental variables and the occurrence of a species can be addressed through correlative or mechanistic approaches [25], and both methodologies have been found to be effective [26,27]. Particularly in the case of plant distribution modeling, correlative approaches have been primarily used to circumvent the difficulty associated to scaling up from physiological attributes to ecosystem level processes [28].

Here, we evaluated exposure to climate change of *Pinus pinaster* Ait. (maritime pine), an ecologically and economically important Mediterranean conifer [29], integrating abiotic and biotic components of exposure. Previous studies have evaluated separately the suitability for the host [30,31] and for the disease [32–36], and only few have assessed suitability in the future [31,35,36]. Abiotic exposure to climate change was assessed by estimating risk of habitat loss in future climate predictions representative of 2050. We incorporated biotic exposure to an emerging disease by considering pitch canker disease, caused by the fungus *Fusarium circinatum* Nirenberg & O’Donnell on *Pinus* species [37], that not only represents a potential biotic threat for *P*. *pinaster* but is also affecting management decisions, e.g. leading managers to the use of broadleaves in plantations. This disease was first detected in Europe in pine nurseries in 2005 [38]. Since then, it has been reported in *P*. *radiata* plantations in Northern Spain [39,40] where it constitutes, together with *P*. *pinaster*, the most abundant species. Although *P*. *pinaster* is only moderately susceptible to pitch canker [41,42], its geographical proximity with the highly susceptible *P*. *radiata* [42], makes this pine likely to become infected by *F*. *circinatum* under high disease pressure. In fact, a pitch canker disease infection within a *P*. *pinaster* plantation in northern Spain has been recently reported [38].

We aim to fulfil the accuracy and resolution requirements needed for local forest management. Consequently, our study area is restricted to the Spanish Iberian Peninsula, for which there are specific sources of information such as data from the Spanish Meteorological Agency (AEMET) and from the Spanish National Forest Inventory. A solid and high resolution assessment of abiotic and biotic exposure of *P*. *pinaster* can assist managers in selecting among the distinct available practices in order to enhance this economically important pine to cope with the aforementioned stresses: breeding programs, particular silvicultural methods [43] and/or different monitoring schemes.

## Materials and methods

### Occurrence data

SDMs require an occurrence dataset including presence records (for all statistical algorithms) and, in general, also absence, pseudo-absence or background records of the target species to base their predictions upon (more details can be found in [23]). However, the difficulty associated to detect real absence records commonly translates into a generalized use of pseudo-absence or background records. In our case, we obtained an occurrence dataset composed of presence and pseudo-absence records for our pine species (*P*. *pinaster*) and pitch canker disease along the study area—the Spanish Iberian Peninsula. In this section, details on the method used for selecting presences are provided. However, as algorithm selection played a major role in defining the number and selection technique of pseudo-absences, these details are thus provided within the SDM section along with other algorithm specifications (more details can be found in [44]).

In the case of *P*. *pinaster*, we used the third Spanish National Forestry Inventory (NFI), developed between 1997–2007 and based on a 1 km grid. This pine is broadly distributed within the Spanish Iberian Peninsula and it has been extensively used in afforestation programs. However, there is no information concerning the fitness of the planted stands or their longevity, or whether they are artificially maintained. Accordingly, to be conservative, we only considered native populations as presences to train a SDM aiming at capturing the relationship between climate and the distribution of the species. In addition, the native distribution of *P*. *pinaster* is also broad and encompasses very different environments, making it thus likely that all different habitats occupied by the species within the Spanish Iberian Peninsula are adequately represented. Accordingly, presences were selected as those plots where natural and seed-born populations of *P*. *pinaster* were reported as one among the three major species, which after removing duplicates, led to a set of 6081 plots. We further eliminated those plots not included within the native distribution of *P*. *pinaster* (assessed from [45]) which reduced the number of selected plots to 2971, and we used the central coordinates of the plots as presence records. The rest of the study area, not fulfilling these criteria, was considered as potential pseudo-absence records.

Concerning pitch canker disease, we used data from a survey performed by different regional authorities in Spain and collected by the Spanish Ministry of Agriculture, Food and Environment in the period 2006–2012. We obtained 159 municipalities where at some point during that period a disease outbreak was declared, located in the north-western side of the Iberian Peninsula. All pitch canker disease outbreaks were reported in *Pinus radiata* D.Don. In order to select adequate coordinates to represent presence records, we divided the study area in a 1 Km grid and selected the 1444 grid cells that were simultaneously (i) within the positive-detected municipality borders and (ii) within the host species’ distribution (*P*. *radiata* obtained from the NFI). Finally, to prevent the municipality size from affecting model outputs (as bigger municipalities tended to have a higher number of disease presences included in the models) we considered a maximum of 10 presences per municipality i.e. the mean number of potential disease presences per municipality. Thus, in those municipalities enclosing less than 10 potential presences all records were included within the definitive presence data set, whereas in those municipalities where potential presences were larger, 10 records were randomly selected to be included within the final presence dataset. The definitive presence dataset reckoned 943 records. Similarly to the case of *P*. *pinaster*, all the rest of the study area was considered as potential pseudo-absence records.

### Bioclimatic data

Global databases do not fulfil the requirements in accuracy or resolution to support local forest management. Indeed, WORDLCLIM [46] has already been reported as problematic in the Spanish Iberian Peninsula (see [47]), as its interpolations are based on 142 clustered meteorological stations, among which the highest altitudes are barely represented (see S1 Fig). To obtain accurate and high-resolution bioclimatic surfaces, we used 5053 meteorological stations with observed daily precipitation data and 1830 with observed daily maximum and minimum temperature data from AEMET, covering the period between 1950–2000 (see S1 Fig for a comparison between WORDLCLIM and AEMET’s meteorological stations). First, monthly variables were calculated as monthly accumulated precipitation and as monthly means for maximum and minimum temperature. Then, we interpolated these monthly variables by means of Thin Plate Splines (TPS) [48] using elevation as independent co-variable to obtain continuous surfaces (1 Km grid cell) across the study area. We selected TPS as the interpolation method, as it has performed well in previous comparative tests of multiple interpolation techniques [49,50], and has been widely used in previous studies—including WORLDCLIM [46] and others such as [51]- and because it is computationally efficient and easy to run. Finally, the 19 bioclimatic variables proposed by WORLDCLIM [46] (BIO1-BIO19; see Table 1) were calculated.

**Table 1:** Complete list of environmental variables tested as candidates to be included in species distribution models (SDMs) for the pitch canker disease and *Pinus pinaster* Ait.

Variable	Explanation	D^2^_*P*. *pinaster*_	D^2^_Pitch canker_
**CLIMATIC VARIABLES**			
BIO1	Annual Mean Temperature	0.12	0.24
BIO2	Mean Diurnal Range: Mean of monthly (max temp—min temp)	0.01	0.43
BIO3	Isothermality (BIO2/BIO7)	0.01	0.32
**BIO4**	**Temperature Seasonality (standard deviation)**	**0.04**	**0.57**
BIO5	Max. Temperature of Warmest Month	0.14	0.50
**BIO6**	**Min. Temperature of Coldest Month**	0.08	**0.24**
BIO7	Temperature Annual Range (BIO5-BIO6)	0.04	0.57
BIO8	Mean Temperature of Wettest Quarter	0.04	0.11
BIO9	Mean Temperature of Driest Quarter	0.08	0.39
BIO10	Mean Temperature of Warmest Quarter	0.15	0.36
BIO11	Mean Temperature of Coldest Quarter	0.10	0.22
**BIO12**	**Annual Precipitation**	**0.06**	**0.49**
BIO13	Precipitation of Wettest Month	0.01	0.42
BIO14	Precipitation of Driest Month	0.13	0.46
BIO15	Precipitation Seasonality (Coefficient of Variation)	0.01	0.07
BIO16	Precipitation of Wettest Quarter	0.01	0.43
**BIO17**	**Precipitation of Driest Quarter**	**0.21**	**0.49**
BIO18	Precipitation of Warmest Quarter	0.19	0.47
BIO19	Precipitation of Coldest Quarter	0.02	0.40
**TOPOGRAPHIC VARIABLES**			
**Elevation**	**Elevation above the sea level (m)**	**0.17**	0.20
Slope		0.01	0.09
**Dist_coast**	**Distance to the coast**	0.04	**0.40**

Note: D^2^ indicates the explained deviance score obtained when individually fitting the variable in a Generalized Linear Model(GLM). Similarly coloured rows group highly correlated variables (Pearson correlation > 0.60), while non-coloured ones indicate non-correlated variables. Variables in bold show the finally selected variables. Climatic variables obtained from AEMET and Topographic Variables from G30TOPO.

As additional variables, we also included distance to the coast (dist_coast) and two topographic variables, namely elevation and slope, both derived from the GTOPO30 model (courtesy of the U.S. Geological Survey), as these have been reported as relevant in determining the distribution of the disease [34,52]. In particular, slope aimed at representing possible soil-related stressors on trees such as soil nutrient content or soil erosion. Table 1 provides for a summary of the variables tested.

In order to avoid multicollinearity effects, variables with Pearson correlations lower than 0.60 were retained (see Table 1 for more information), as the use of simple methods based on rules of thumb has proved to be as effective as more complicated methods [53]. Among highly correlated variables we kept the one with highest explained deviance scores (D^2^) when individually fitted in a Generalized Linear Model (GLM—[54]). We avoided the use of BIO8 and BIO9, because the steep gradient shown by these variables, in which very often two adjacent cells are characterized by extremely different values within the study area for no obvious reason, may lead to artefacts in the SDM output maps. Finally, possible collinearity problems were circumvented by performing a Variance Inflation Factor (VIF—[55,56]), ensuring that all VIF values were below 5. The final sets of relevant weakly correlated variables to build SDMs were (i) BIO4—Temperature Seasonality, BIO12—Annual Precipitation, BIO17—Precipitation of Driest Quarter and elevation for *P*. *pinaster* and, (ii) BIO4—Temperature Seasonality, BIO6—Mean Temperature of Coldest Month, BIO12—Annual Precipitation, BIO17—Precipitation of Driest Quarter and Distance to the coast for the disease. For both pine and disease, mean and standard deviation values of the environmental variables were also calculated separately for presences and absences to get insights about their currently inhabited habitat conditions.

Future climatic variables (monthly accumulated precipitation and monthly maximum and minimum temperature) representative of 2050 (average for 2041–2060) were obtained from nine of the most recent Global Climate Models (GCMs) used in the Intergovernmental Panel on Climate Change (IPCC) Fifth Assessment report [4] (see Table 2). As future projection scenarios, we used two different Representative Concentration Pathways (RCP) namely RCP4.5 (medium emission scenario) and RCP8.5 (high emission scenario) [57]. Thus, the combination of 9 GCMs and two different scenarios (RCP4.5 and RCP8.5) led to a total of 18 different future climate predictions. These climatic predictions were combined and used together through this study (see Future suitability maps section) to assess abiotic and biotic exposure on *P*. *pinaster*.

**Table 2:** Global Climate Models (GCMs) used to obtain future climate predictions representative of 2050. All GCMs were calculated for two different Representative Concentration Pathways (RCPs), namely RCP4.5 and RCP8.5, totalling to 18 (9 x 2) different future climate predictions.

Model	Institution	Country	Resolution (lon × lat)
BCC-CSM1-1	Beijing Climate Centre (BCC), China Meteorological Administration	China	2·8 × 2·8°
CanESM2	Canadian Centre for Climate Modelling and Analysis (CC-CMA)	Canada	2·8 × 2·8°
CNRM-CM5	Centre National de Recherches Meteorologiques/Centre Europeen de Recherche et Formation Avancees en Calcul Scientifique (CNRM-CERFACS)	France	1·4 × 1·4°
GFDL-ESM2 M	Geophysical Fluid Dynamics Laboratory (GFDL)	United States	2 × 2·5°
HADGEM2-CC	Met Office Hadley Centre (MOHC)	UK	1·87 × 1·25°
MIROC-ESM-CHEM	Japan Agency for Marine-Earth Science and Technology (JAMSTEC), Atmosphere and Ocean Research Institute (AORI), and National Institute for Environmental Studies (NIES)	Japan	2·8 × 2·8°
MPI-ESM-MR	Max Planck Institute for Meteorology (MPI-M)	Germany	1·8 × 1·8°
MRI-CGCM3	Meteorological Research Institute (MRI)	Japan	1·2 × 1·2°
NorESM1-M	Norwegian Climate Centre (NCC)	Norway	2·5 × 1·9°

GCMs, which have a coarse resolution (*ca*. 2 degrees), were transformed to a local scale using a two-step analogue statistical downscaling method developed by [58]. The first step is an analogue approach [59] in which the *n* most similar days to the day to be downscaled are selected by using four different meteorological large-scale fields. The second step differed depending on the target variable: (i) precipitation was calculated by re-assigning the calculated amounts using an empirical distribution function; and (ii) temperature was obtained by using a multiple linear regression analysis using the *n* most analogous days selected. We followed this procedure for each AEMET meteorological station and for each target day, and then averaged the resulting daily simulations to obtain monthly means. Further details of the methodology are described in [58]. A systematic error was obtained when comparing the simulated data from climate models with the observed data from reference time series due to the inherent downscaling methodology error and to the inner GCM error (which usually incorporates a bias over the data). Thus, to improve our simulations, future climate projections were corrected according to a parametric quantile–quantile method [60]. Then, the obtained variables (future monthly accumulated precipitation and future monthly maximum and minimum temperatures) were interpolated across the entire study area following the same procedure as employed in current climate interpolations (TPS). Finally, we calculated the set of selected WORLDCLIM bioclimatic variables representative of 2050 namely BIO4, BIO6, BIO12, BIO17. We assumed that Distance to the coast and Elevation, remained constant within the analyzed period (from now to 2050)

### Species distribution models

We used generalized additive models (GAM—[61]) to capture the relationship between climate and species’ occurrence records. This algorithm has been widely used in the literature [9,15,62,63], as it allows mimicking different types of response curves using a nonparametric smoothing function instead of parametric terms, providing a better alternative than most other widely-used models such as generalized linear models or classification tree analysis [64,65]. Models were processed in BIOMOD [66] using the package “biomod2” (default settings for the model) in the R statistical software environment [67]. As recommended for GAM in [44], we randomly selected 10000 pseudo-absences, and same weight was given to presences and absences. Finally, a five independent 70–30 training-evaluating subsets of the data for model evaluation was performed, and model performance was assessed as the mean True Skill Statistic (TSS—[68]). Based on the model trained on current climate data, we obtained for both, the pine and the disease, a probabilistic prediction of habitat suitability for each climatic data set (current climate and 18 future climate predictions). These were converted to binary suitability maps by defining probability thresholds that maximized TSS values. Thereby, we obtained 19 binary projections, one corresponding to current climate and the remaining 18 to the different future climate predictions tested.

Variable importance was analysed following the method available in the package “biomod2”: First, the model was trained with the selected environmental variables and predictions were performed (reference predictions). Then, one environmental variable was changed to random values and new predictions were done. This process was repeated four times with each environmental variable included in the model. Finally, we calculated Pearson´s correlations—which range between 0 (no correlation) and 1 (maximum correlation)—between reference predictions and the new ones. The final score for each environmental variable is provided as the difference between 1 and mean correlation among the four repetitions performed for each variable so that higher values indicate higher importance.

### Future suitability maps

For the host and the disease separately, we obtained 18 future binary projections which classified each grid cell of the study area as suitable or unsuitable in 2050. In order to incorporate the uncertainty derived from the wide range of future climate predictions available avoiding the use of an average prediction, we followed the methodology proposed in [31]. According to their work, the number of future climate predictions projecting suitability in one specific cell was used as an indicator of the degree of agreement among models about the future habitat being suitable in that cell. Thus, the 18 binary projections were combined resulting in a map in which values could possibly range from 0 (none of the future climate predictions was projected to be suitable) to 1.0 (all future climate predictions tested were projected to be suitable) with higher scores indicating higher agreement of suitable habitat in the future. Finally, as proposed in [31], three different future suitability categories were defined as: “likely suitable” with suitability scores >0.7 (suitable habitat for more than 70% of future projections), “uncertain” with a suitability score of 0.36–0.7, and “likely unsuitable” with suitability scores <0.36. We performed this analysis for each species individually.

### Abiotic and biotic exposure for *P*. *pinaster* Ait.

Abiotic and biotic exposures of *P*. *pinaster* were assessed by means of current and future suitability maps (both for *P*. *pinaster* and pitch canker disease respectively). Particularly, when assessing future exposure, we used the three different suitability categories defined in the future suitability maps,—i.e. “likely suitable”, “uncertain” and “likely unsuitable”- as the basis for recommending the most appropriate management to facilitate populations’ ability to cope with climate change. For this analysis, we considered the entire distribution of the species *P*. *pinaster*, including both native and planted populations, and the results were presented following the Spanish seed zones of forest reproductive material [69]. Seed zones delimit ecologically homogeneous distribution areas and, thus, are expected to group genetically similar populations likely to be locally adapted, as well as to differ in their productivity, and thereby in their economic impact on regional economies. Consequently, seed zones are appropriate as management units and provide a perfect framework to guide forest management.

To assess future abiotic exposure, we employed *P*. *pinaster*’s future suitability map. Likely suitable areas highlighted locations barely exposed where no special management for adaptation to climate change was needed. Contrarily, likely unsuitable areas indicated high abiotic exposure sites where additional measures should be considered such as breeding programs or silvicultural actions enhancing the species’ capacity to cope with climate change. Furthermore, the future suitability map of the disease was used to assess future biotic exposure. Likely suitable locations for pitch canker were considered as high biotic exposure areas where urgent actions were needed to improve the capacities of *P*. *pinaster* to avoid possible infections, while likely unsuitable locations were considered as low biotic exposure areas where no further actions were needed. Finally, uncertain areas in both future suitability maps indicated locations where there was not an agreement among future climate predictions and, thus, where monitoring is needed in order to see the development of populations and to address its management accordingly.

## Results

### Bioclimatic data and species distribution models

We found few differences between the mean environmental conditions in presences and pseudo-absences records of *P*. *pinaster* (see Table 3 and S2a Fig), an outcome that was expected given the low explained deviance scores obtained by most of its environmental predictors (see Table 1). Nevertheless, this analysis only considered environmental variables individually, and the SDM fitted for *P*. *pinaster*, which considers altogether the set of selected environmental variables, had an acceptable performance as revealed by its TSS score (0.69) and its sensitivity and specificity values (93.50 and 75.30 respectively).

**Table 3:** Mean values and variable importance, calculated with the package “biomod2” in R statistical software, of the environmental variables used to fit species distribution models for *Pinus pinaster* and pitch canker disease. Standard deviation is shown in brackets.

Variable	Mean				Variable Importance	
	*P*. *pinaster*		*Pitch canker*		*P*. *pinaster*	*Pitch canker*
	Presences	Absences	Presences	Absences		
BIO4	638.7 (51.3)	617.8 (84.1)	428.2 (35.9)	618.3 (84.6)	0.19	0.56
BIO6 (°C)	-	-	3.4 (1.1)	1.1 (2.5)	-	0.36
BIO12 (mm)	694.9 (223.2)	694.2 (345.4)	1452.9 (225.0)	693.2 (341.9)	0.11	0.46
BIO17 (mm)	80.0 (17.3)	81.6 (51.23)	199.2 (46.1)	81.5 (51.2)	0.50	0.45
Dist. Coast (Km)	-	-	17366.3 (12613.8)	129052.6 (86349.8)	-	0.40
Elevation (m)	968.8 (240.6)	685.84 (396.5)	-	-	0.25	-

**Fig 1:**
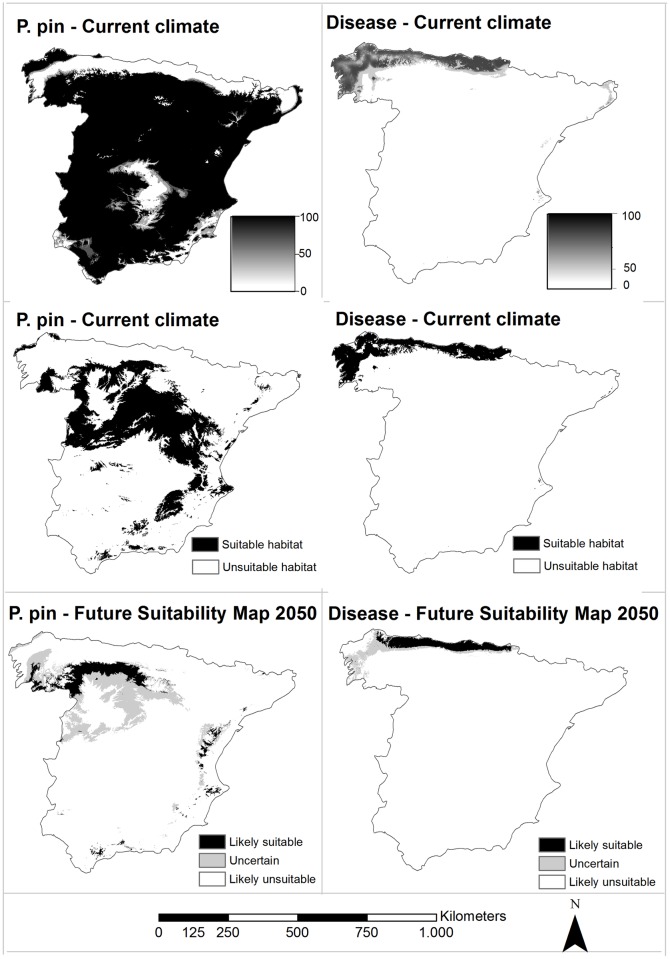
Geographic projections of species distribution models of *Pinus pinaster* (P. pin; a-c) and pitch canker disease (Disease; d-f). Current climate projections are shown in probabilistic projections, where the values oscillate between 0 to 100—a) and d)—and in binary projections, restricted to 0 or 1 values—b) and e). Future suitability maps summarizing 18 future climate predictions are shown in c) and f).

By contrast, large differentiation between the mean environment of presences and pseudo-absences was found in pitch canker disease (see Table 3 and S2b Fig). The distribution of the disease seemed to be constrained by low temperatures (represented by BIO6) as well as by low precipitation regimes (BIO12 and BIO17; see Table 3 and S2b Fig). Short distance to the coast was also found to be very relevant for the disease’s habitat suitability, presumably due to the higher relative humidity in these locations, a key factor during the infection stage [70,71]. The SDM of the disease performed very well as assessed by its evaluation scores (TSS = 0.93, sensitivity = 99.26 and specificity = 93.63), revealing a very important role of climate in determining the distribution of the disease. The detected suitable area was located along the north-western side of the Iberian Peninsula (see Fig 1d and 1e), which is consistent with the declared infection outbreaks in Spain. We also detected marginal suitability areas along the eastern coast of the Iberian Peninsula, where there are no declared infection outbreaks.

Pseudo-absences are ultimately representing the entire study area so they are very similar for the pine and the disease (see Table 3 and S2 Fig). All variables included in the SDMs were highly significant for both the pine and the disease (p-value < 0.0001). As for variable importance (see Table 3), BIO17 obtained by far the highest score in the SDM of *P*. *pinaster* as compared to the other variables included in the model. Variables in the SDM of the disease obtained more balanced scores than in the case of *P*. *pinaster*, although BIO4 was the most influential variable. Finally, the threshold values maximizing TSS, and thus employed for transforming probabilistic into binary projections, were 60.5 and 45.0 respectively for *P*. *pinaster* and pitch canker disease.

### Future suitability maps

Future suitability maps revealed a reduction in suitable territory for both the pine and the disease (see Fig 1c and 1f). In the case of *P*. *pinaster*, the reduction in habitat suitability was more severe along the central and south-western Iberian Peninsula, as most of the likely suitable territory concentrated in the central plateau (seed zones 5, 16 and 17; comparison between Fig 1b and 1c). Concerning pitch canker disease, the reduction in habitat suitability mainly occurred in the north-western edge of the study area (seed zones 1 and 2), while the rest of likely suitable areas was very similar to that detected as currently suitable for the species (comparison between Fig 1e and 1f).

### Abiotic and biotic exposure for *P*. *pinaster* Ait.

**Fig 2:**
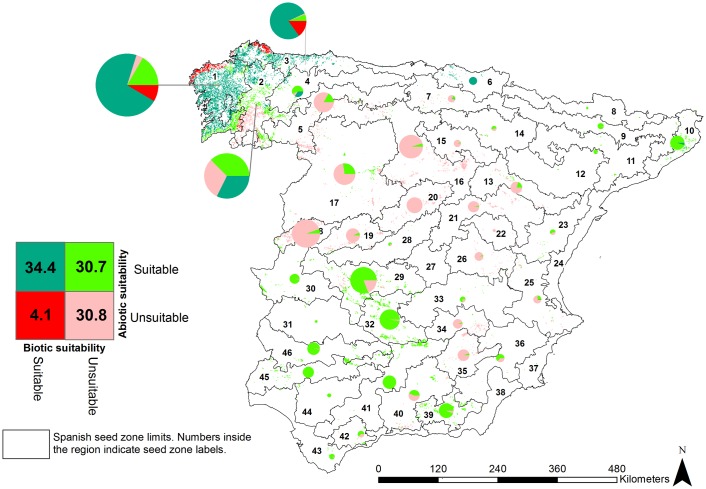
Current abiotic and biotic exposure assessment of *Pinus pinaster* Ait approached by current suitability maps of *P*. *pinaster* and pitch canker disease respectively. Charts sizes are proportional to *P*. *pinaster* occupancy within deployment regions. Numbers in the legend correspond to the percent of the distribution classified within each exposure combination.

**Fig 3:**
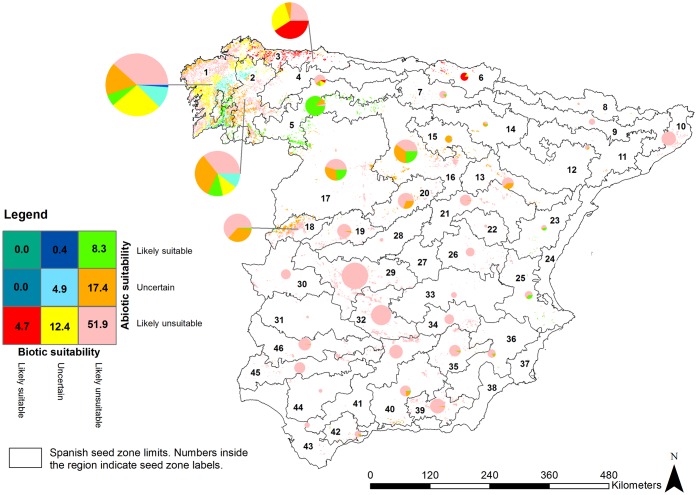
Future (2050) abiotic and biotic exposure assessment of *Pinus pinaster* Ait approached by future suitability maps of *P*. *pinaster* and pitch canker disease respectively. Charts sizes are proportional to *P*. *pinaster* occupancy within deployment regions. Numbers in the legend correspond to the percent of the distribution classified within each exposure combination.

We assessed exposure across all the populations of *P*. *pinaster* within the study area depending on the current and future suitability maps of the host and the disease (see Figs 2 and 3).

Overall, the exposure undergone by *P*. *pinaster* is largely increased in future climate scenarios, as the percentage of non-exposed populations decreases from 30.7% in current climate to 8.3% then (see Figs 2 and 3). However, this is mainly due to a higher abiotic exposure, as the rate of populations under both abiotic and biotic exposure is maintained between both periods. Our results point to a reduction in biotic exposure that is particularly important in the north-western side of the Iberian Peninsula (seed zones 1, 2 and 3; comparison Figs 2 and 3).

When looking into more details at future exposure, all populations composing the distribution of *P*. *pinaster* are exposed to a certain degree (but for ca. 8% of the distribution, concentrated around the central plateau and north-western edge of the study area, seed zones number 1, 2, 5, 16 and 17, which were classified as low exposure for both, abiotic and biotic components). Nevertheless, only circa 5% of the distribution of *P*. *pinaster* was classified as highly exposed to both abiotic and biotic factors. These populations were located in the northernmost distribution of the species, mostly in the populations from seed zones 3 and 6 (see Fig 3).

Abiotic exposure affected *P*. *pinaster* across its entire distribution: over 50% of its distribution was classified as highly exposed to abiotic factors. In contrast, areas with high exposure to pitch canker concentrated in the northern edge of the study area, which translated into almost 80% of the distribution classified to be under low biotic exposure.

## Discussion

Overall, the entire distribution of *P*. *pinaster* in the Spanish Iberian Peninsula is affected by abiotic exposure, meaning that predicted climatic shifts result in a large reduction of its environmental suitability across the study area (but for some exceptions—see seed zones 1, 2, 5, 16 and 17). Abiotic exposure seems to be particularly important along the central and southernmost edge of the Iberian Peninsula, as all populations in these locations are highly exposed. This result is consistent with those obtained in a previous work showing that the genetic groups of *P*. *pinaster* located along the Spanish Mediterranean coast were considerably threatened by climate change [31]. These locations correspond to the rear edge of the distribution of the species and are prone to suffer more strongly the consequences of climate change in comparison to others located at northern locations in Europe [72]. Abiotic exposure is likely to be a consequence of the predicted increased intensity and/or duration of droughts (Mediterranean mid-latitudes; [4]), as suggested by the importance of BIO17 (Precipitation of Driest Quarter) in determining the species’ distribution (see Tables 1 & 2). In fact, variables representing drought were also relevant in determining the distribution of the pine in previous studies including its entire range [30,31] and in other local studies [43,73].

Current suitable locations for the disease concentrated in the north-central and north-western edge of the study area, supporting previous studies predicting the potential global distribution of pitch canker disease in *Pinus* spp [33]. Furthermore, we found that future climatic changes are likely to constrain its distribution to the north of the Iberian Peninsula (mostly in seed zones 3 and 6), thus reducing biotic exposure on the north western pine populations (seed zones 1 and 2). This outcome is probably linked to the expected reduction in precipitation [4], which plays a major role in the distribution of the disease (see S2 Fig).

Management aiming at favouring adaptation processes in forest ecosystems encompasses three different strategies, namely (i) breeding programs incorporating new and adapted genes; (ii) silvicultural methods directed to enhance local adaptation; and (iii) management strategies to prevent disease occurrence. Selecting one of them should directly depend on exposure and on the importance of the target population on the regional economies. When deciding among these, exposure assessment, together with productivity, play a fundamental role. Forest management of *P*. *pinaster* would benefit from strategies directed at improving the species’ capacities to deal with drought stresses as well as at enhancing the species’ resistance to *Fusarium circinatum*. In this direction, breeding programs are likely to be effective, as differences in responses to drought and to the pathogen have been reported to be genetically controlled [74,75]. In fact, in a provenance/progeny trial [41], large differences in resistance to pitch canker disease were found among and within populations suggesting a host response to the disease through natural or artificial selection, with important genetic gain for *P*. *pinaster* resistance. Nevertheless, because breeding programs are expensive and long lasting, they should be applied to specific, highly-exposed and valuable populations with impact on regional economies, such as those located at the northernmost edge of the distribution [76], which coincide overall with areas under high biotic exposure (seed zones 1 to 6). In addition, areas under uncertain biotic exposure (particularly those from seed zones 1 and 2) should be closely monitored, given the potential consequences of a disease outbreak, and that a modification in the dynamic of the host-pathogen interaction could result in significant changes in the predicted impact of the disease. The remaining populations are mostly affected by abiotic exposure, so a monitoring program should be applied to detect changes either in the host or the pathogen, and silvicultural practices aimed to fasten adaptation. Among these practices, those directed at reducing competition for (water) resources, such as decreasing tree density [43] and favouring unevenness between sizes and ages of the trees in a stand [73], are particularly adequate for *P*. *pinaster*. In addition, thinning [43] and extending rotation times—as juveniles are more sensitive to droughts than adults [73]–are other recommended management guidelines for threatened populations.

The *P*. *pinaster* exposure maps do not take into consideration other non-climatic variables that can play a role in the future distribution of the species, such as local adaptation and phenotypic plasticity [77,78], or migration [79,80]. In addition, other factors like soils, anthropogenic interactions or intraspecific competition, could have played a role in determining the distribution of the pine, but mainly in specific areas (Fig 1a and 1b), where the suitable habitat extended largely beyond the current distribution. For now, the integration of additional explanatory variables in our models is limited by the aforementioned lack of empirical data, and also in relation to the host-disease dynamic. Moreover, although incorporating a wide range of GCMs (9) and scenarios (2) in SDMs has enabled us to reduce the uncertainty derived from the wide range of future climate predictions, there are additional sources of uncertainty, e.g. the one derived from algorithm selection [31]. However, our models succeed in assessing the relationship between climate and the pine’s distribution in order to evaluate whether its current populations are likely to be subjected to climatic stress in the future (see [81] for more details on the climatic niche).

Other considerations to take into account in our models are that future suitability maps rely on the assumption of niche conservation along a 30-year timespan (i.e. a constant relationship between each species and climate). In the case of *P*. *pinaster* this is foreseeable, as it is a long-lived species with long rotation times (from 30 to almost 100 years) and, thus, involving only one generation during this period. Furthermore, although *F*. *circinatum* reproduces asexually [82], Spanish clonal populations are composed of two widely distributed genotypes [82], and it is unknown how climate change will influence the growth and reproduction of any of them and their interaction with the host.

The suitability maps of pitch canker disease can be used to assess biotic exposure of any other (pine) species affected by it, although its incidence or the severity of it would vary according to species susceptibility. In fact, for this study, we trained our models based on infections detected in *P*. *radiata* although there is some evidence indicating that *P*. *pinaster* is more resistant to the infection, as shown by the progress curve of the area under disease (AUDPC) [83], and the mean wound size [42]. Furthermore, procuring a consistent model of pitch canker disease is challenging given the difficulty associated to obtain a reliable occurrence dataset. In addition, other factors not related to climate, e.g. distribution of the host species, different pine species resistance to the disease and effective prevention plans, also influence the current distribution of the disease.

Our work, illustrated with *P*. *pinaster*, provides an approach to assess abiotic and biotic exposure with regards to climate change. Nevertheless, other factors could be considered to better characterize the exposure of the species to climate change. For instance, fire should be incorporated as an additional abiotic factor, as it plays an important role in pine biology [29,84], while other pests, such as the pine processionary moth (*Thaumetopoea pityocampa* Den. & Schiff. [85]) or the pine weevil (*Hylobius abietis* L. [86]), should also be included.

Finally, we contribute to forest management in three ways: (i) our pitch canker future suitability map can be employed to assess biotic exposure of any other susceptible species in the Spanish Iberian Peninsula; (ii) we provide a high resolution and scientifically solid assessment of exposure for *P*. *pinaster* in the Spanish Iberian Peninsula that can be directly employed in delineating its breeding and management programmes; and (iii) our approach can be easily transferred to any other species.

## Supporting information

S1 Fig Geographical representation and histogram of elevations of the meteorological stations from AEMET and WORLDCLIM. (DOCX)

S2 Fig Selected environmental variables for Pinus pinaster Ait. (a) and pitch canker disease (b) to be included within their species distribution models. (DOCX)
