# Supplementary material for: Incorporating exposure to pitch canker disease to support management decisions of Pinus pinaster Ait. in the face of climate change
Source: PLoS One. 2017 Feb 13;12(2):e0171549. doi: 10.1371/journal.pone.0171549 (PMC5305074; doi:10.1371/journal.pone.0171549)
Supplement: S1 Fig — (DOCX) [file pone.0171549.s001.docx]

b)

a)


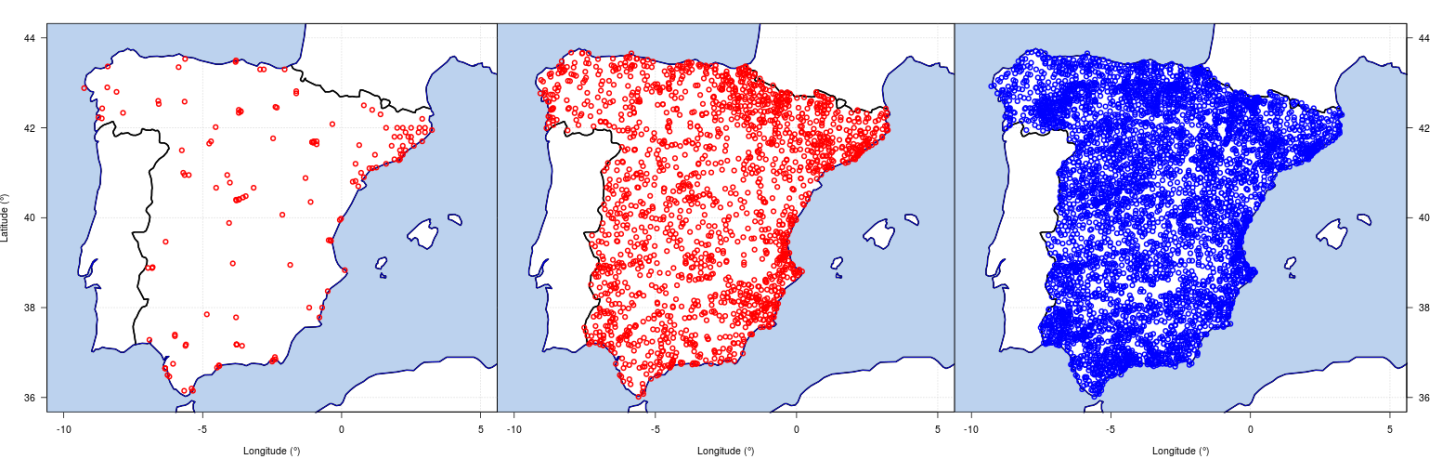


S1 Fig: Geographical representation (a) and histogram of elevations (b) of the 142 meteorological stations employed for climatic interpolations in WORLDCLIM database (temperature and precipitation; Hijmans et al., 2005) and the 1830 (temperature) and 5053 (precipitation) meteorological stations provided by the Spanish Meteorological Agency (AEMET) across the Spanish Iberian Peninsula.

**WORLDCLIM – Temperature and Precipitation**

**AEMET - Temperature**

**AEMET - Precipitation**


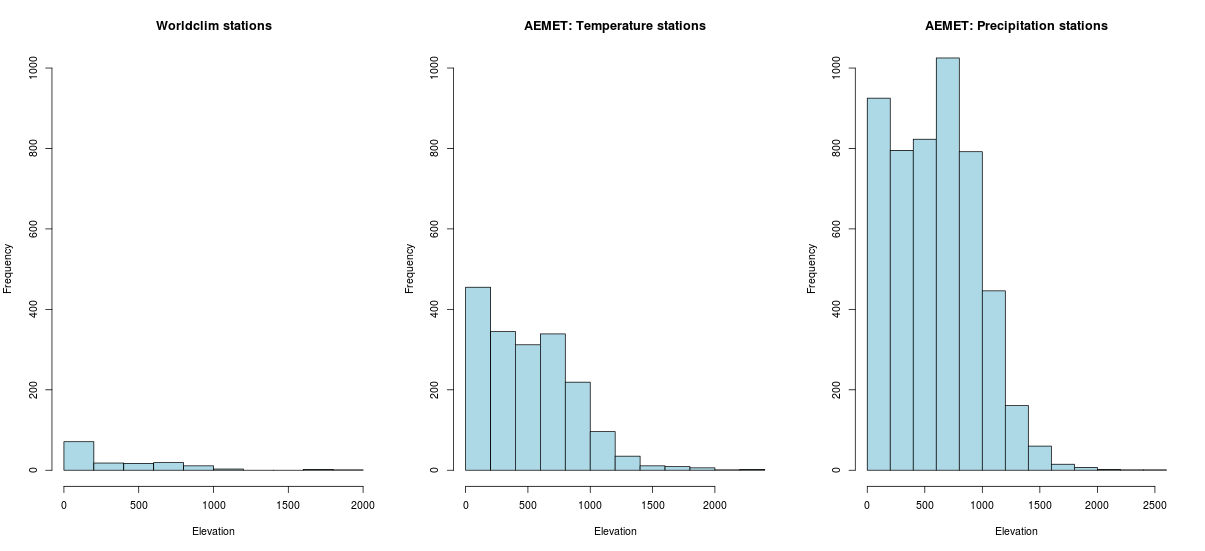


**WORLDCLIM – Temperature and Precipitation**

**AEMET - Temperature**

**AEMET - Precipitation**
