# Supplementary material for: Incorporating exposure to pitch canker disease to support management decisions of Pinus pinaster Ait. in the face of climate change
Source: PLoS One. 2017 Feb 13;12(2):e0171549. doi: 10.1371/journal.pone.0171549 (PMC5305074; doi:10.1371/journal.pone.0171549)
Supplement: S2 Fig — (DOCX) [file pone.0171549.s002.docx]

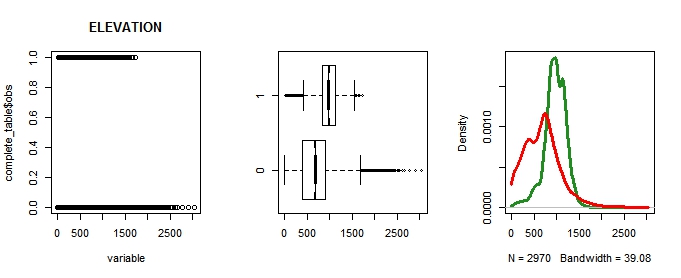

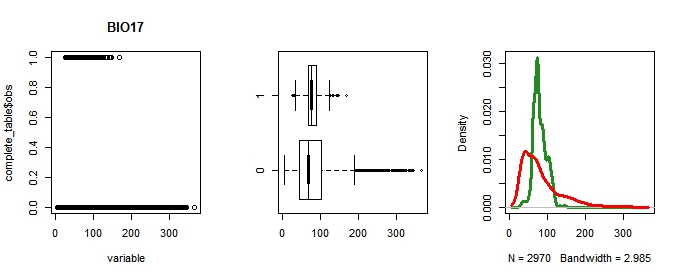

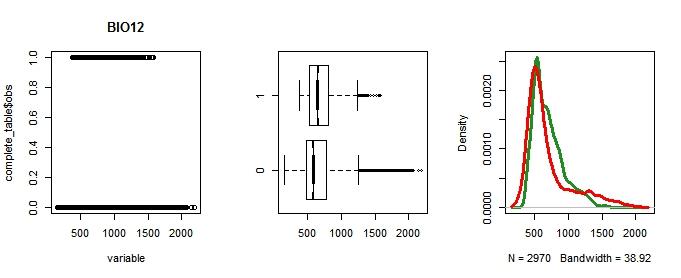

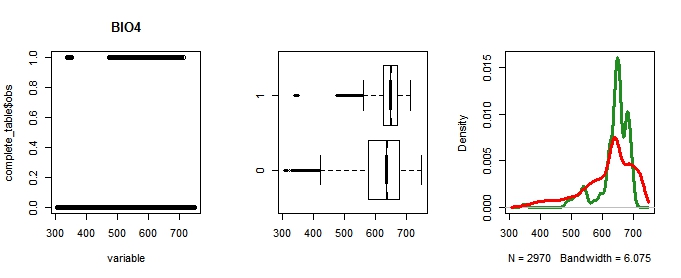


a)


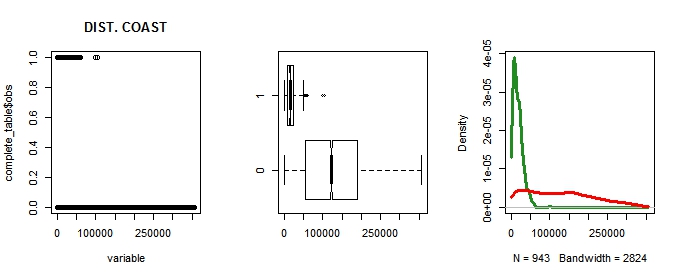

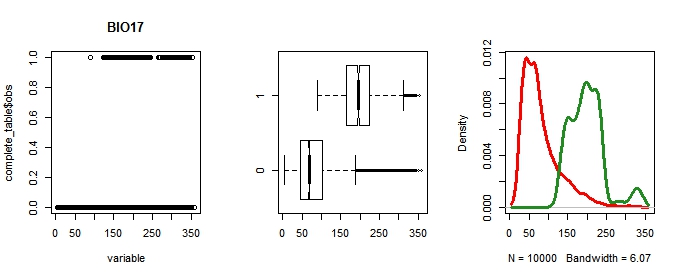

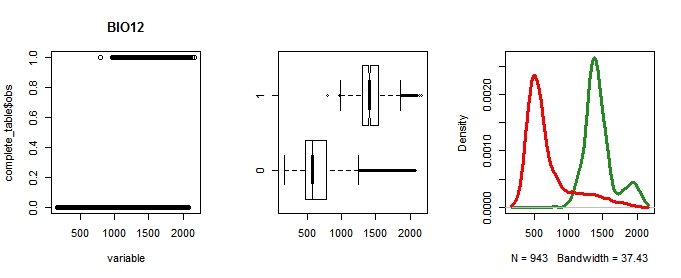

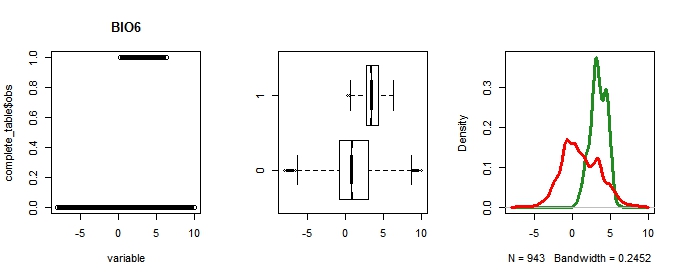

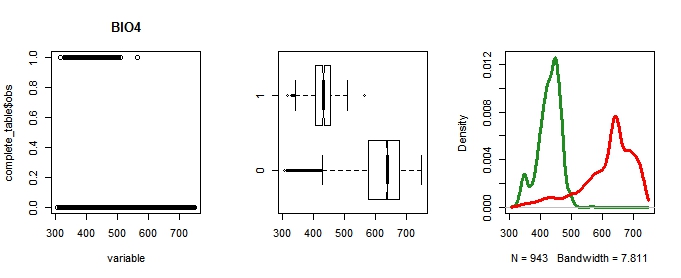


b)

Fig S2: Selected environmenal variables for *Pinus pinaster* Ait. (a) and pitch canker disease (b) to be included within their species distribution models. 1/0 values as well as green and red lines correspond to presence and pseudoabsence records respectively.
